# Frequency of Dental Caries in Children

**Published:** 1898-12

**Authors:** B. J. Cigrand


ARTICLE V.

### FREQUENCY OF DENTAL CARIES IN CHILDREN.

Dr. M. Lipschitz, of Berlin, gave a most interesting paper on the above subject before the recent Congress at Moscow. It demonstrates as no paper of the past has, that dental caries is a disease frequently met with in the youthful population of Europe; it establishes the fact that this human ailment is abroad and that the impression that "in Europe the people have little difficulty with dental caries" is wrong. Among other things he says: "The great scope of dental caries is well known to the dental operator, but it is within the last few years that anything has been done to found our knowledge of this matter on statistics. In order to ascertain whether or not the frequency of dental caries is deserving of national attention, it was necessary to demonstrate by means of reliable statistics that the human economy is heir to no greater disease than dental caries. It was suggested and put into effect that a systematic investigation of the condition of the teeth of school-children be inaugurated, the investigation to be conducted in the several large cities of Germany, and if possible to extend to foreign lands.

In 1891, as near as can be determined, the first report relative to dental caries among school children was put into print. In Lucern, Switzerland, 1,000 children, ranging from 7 to 14 years of age, showed 94 per cent. suffering from caries. In Germany Herr Feuchel in 1893 examined 335 pupils with this result : 12 presented perfect dentures; 200 boys 97 per cent. decayed teeth, 135 girls 95 per cent. These figures were gotten at the poor-house at Hamburg, and the results of his investigation led him to probe into the condition of teeth at schools and academies which had pupils from more hygienic quarters. Among 693 children, 98 per cent of the girls presented decayed teeth and 99 per cent. of the boys.

Roese, who gave even greater attention to this matter, published the following from the schools of Freiburg: Among 3,460 children, 98.7 per cent. suffered from dental caries; and from neighboring towns 747 children showed 98 per cent.

Berton observed among 3,347 gathered from 21 villages that th eboys suffered less than the girls, the former 81.3 per cent., the latter 84.6 per cent. He examined 78,348 teeth, and of this number 15.3 per cent. were decayed.

In Ungarn Herr Unghvari noted 87.2 per cent of 1,000 children afflicted with dental caries, and in 3,961 teeth 15.4 per cent. were decayed.

In England the British Dental Association under the direction of Dr. George Cunningham examined 10,517 mouths, presenting 76.78 per cent to 94.5 per cent. At one time after examining 40,000 teeth 4 were found with fillings; at another time 100,000 presented 237 fillings.

In Sweden under the direction of Foeberg (1897), in 1,617 children 97.2 per cent. had decayed teeth. In the Masonic Orphans' Home 117 children showed 93.1 per cent. afflicted with dental caries.

My (Lipschitz) own observations among 467 children (girls) of one of the Berlin schools gave the following results : 339 suffered from dental caries. I am of the opinion that great good will come from these investigations, and I would advocate that dental societies throughout the civilized world give this matter consideration and make reports of discoveries—Translated by Dr. B. J. Cigrand from "Deutsche Montsschrift fuer Zahnheilkunde, Oct. 1897.

